# Prevalence of *Enterobius vermicularis* among preschool children in 2003 and 2013 in Xinxiang city, Henan province, Central China

**DOI:** 10.1051/parasite/2016030

**Published:** 2016-07-26

**Authors:** Shuai Wang, Zhijun Yao, Yichen Hou, Dong Wang, Haizhu Zhang, Jingbo Ma, Luwen Zhang, Shiguo Liu

**Affiliations:** 1 Department of Human Parasitology, School of Basic Medical Sciences, Xinxiang Medical University Xinxiang Henan 453003 PR China; 2 Class–2012 Grade of Department of Anesthesiology, Medical College of Nanchang University Nanchang Jiangxi 330006 PR China

**Keywords:** *Enterobius vermicularis*, Enterobiasis, Prevalence, Preschool children, Central China

## Abstract

The present study was performed to assess the prevalence of *Enterobius vermicularis* infection among preschool children in Xinxiang city, Henan province, China and the changes in the egg positive rate for *E. vermicularis* over a 10 year period. A total of 510 preschool children in 17 kindergartens were examined using the cellophane-tape perianal swab method in 2003, while 1734 preschool children in 10 kindergartens were examined in 2013 using the same method. The overall egg positive rate for *E. vermicularis* was 12.75% (65 out of 510) in 2003 and 5.13% (89 out of 1734) in 2013; the former was significantly higher than the latter (*p* < 0.05). In both 2003 and 2013, the egg positive rate for 5 to 6-year-old children was significantly higher than that of 2 to 4-year-old children (*p* < 0.05). However, positive rates were not significantly dependent on gender or area. Among selected personal hygiene factors, no hand washing before eating, sucking fingers or toys, and scratching around the anus were all associated with enterobiasis. The present study confirmed that the prevalence of *E. vermicularis* infection among preschool children decreased significantly over the 10 year period in Xinxiang city, but infection was still prevalent. Improving sanitation and personal hygiene practices, especially hand washing, could help prevent the transmission of *E. vermicularis*.

## Abstract (fr)

La présente étude a été réalisée pour évaluer la prévalence de l’infection par *Enterobius vermicularis* chez les enfants d’âge préscolaire dans la ville de Xinxiang, province du Henan, en Chine et les changements dans le taux de positivité pour oeufs d’*E. vermicularis* sur une période de dix ans. Un total de 510 enfants d’âge préscolaire dans 17 jardins d’enfants ont été examinés à l’aide de la méthode de prélèvement périanal par bande de cellophane en 2003, tandis que 1734 enfants d’âge préscolaire dans 10 jardins d’enfant sont été examinés en 2013 en utilisant la même méthode. Le taux global de positivité pour œufs d’*E. vermicularis* était de 12.75 % (65 sur 510) en 2003 et 5.13 % (89 sur 1734) en 2013, le premier étant significativement plus élevé que le second (*p* < 0.05). En 2003 et 2013, le taux de positivité d’œufs des enfants de 5 à 6 ans était significativement plus élevé que pour les enfants de 2 à 4 ans (*p* < 0.05). Cependant, les taux n’étaient pas significativement dépendants du sexe et du quartier. Parmi les facteurs d’hygiène personnelle sélectionnés, l’absence de lavage des mains avant de manger, sucer les doigts ou des jouets, se gratter autour de l’anus ont été tous associés à l’oxyurose. La présente étude a confirmé que la prévalence de l’infection à *E. vermicularis* des enfants d’âge préscolaire a diminué de façon significative au cours des dix dernières années à Xinxiang, mais l’infection est encore répandue. Améliorer l’assainissement et la pratique de l’hygiène personnelle, en particulier le lavage des mains, pourrait aider à prévenir la transmission d’*E. vermicularis*.

## Introduction

Enterobiasis is a nematode infection caused by the pinworm, *Enterobius vermicularis* [11]. The principal mode of transmission of *E. vermicularis* is direct contact between infected and uninfected persons [16]. For this reason, it is usually endemic in overcrowded conditions, such as kindergartens and primary schools, due to easy transmission from infected to uninfected children [12].

Enterobiasis is asymptomatic in most adults who have low worm burdens. However, in children, particularly those with high worm burdens, neurological symptoms including nervousness, restlessness, irritability, and distraction may occur, and these may influence child growth [13, 14, 18].

*E. vermicularis* infection is prevalent throughout the world, including in developed countries [4, 8, 13, 14, 18], and it is estimated that 4–28% of children are infected globally [1]. *E. vermicularis* infection is also widespread among preschool children in the People’s Republic of China (China). According to the 2011 national parasitic survey, the average prevalence of *E. vermicularis* infection in children reached 17.8% in China, with the highest prevalence rates in Hainan (51.1%), Guangxi (26.7%), and Guangdong (26.7%) [2]. In 2011, the overall prevalence of *E. vermicularis* infection reached 54.86% in Gaozhou city, Guangdong province, southern China [12].

Currently, little information is available regarding the prevalence and possible risk factors of enterobiasis among kindergarten children in the central provinces of China. Therefore, in this study, we evaluated the prevalence of *E. vermicularis* among kindergarten children in Xinxiang city, Henan province, a province of central China, and performed a comparative analysis of the state of *E. vermicularis* prevalence in children in the years 2003 and 2013.

## Materials and methods

### Ethics statement

The study was reviewed and approved by the Ethics Review Committee of the Xinxiang Medical University (Reference Nos. 2003008 and 2013012). All examinations were carried out with permission from the preschool teachers and the children’s parents.

### Subject recruitment

The study was conducted in Xinxiang city, Henan province, central China. A total of 510 preschool children (282 boys and 228 girls) in 17 kindergartens were recruited for the survey in 2003, while 1734 preschool children (918 boys and 816 girls) in 10 kindergartens were recruited in 2013. Seven of the selected 17 kindergartens in 2003 merged with the 10 remaining kindergartens before the year 2013. We therefore recruited preschool children from the 10 remaining kindergartens in 2013. After 10 years of development, the scale of enrollment of the 10 kindergartens expanded, along with the increase in the number of classrooms and teachers in these schools. The average number of teachers in each class increased from two to three. Conversely, the average number of students in each class decreased from 35 to 30. The infrastructures of kindergartens, such as toilets and playground equipment, are much cleaner and more spacious than 10 years ago.

### Questionnaire survey

The questionnaire included questions on basic information regarding the children and their personal hygiene habits (see Table 2). Personal data included age, gender, and residential district. The children’s personal hygiene habits section aimed to gather information on certain behaviors, including hand washing before eating, sucking fingers (including fingernail biting) or toys, and scratching around the anus.

### Detection of *E. vermicularis* infection

Egg detection for *E. vermicularis* was performed using the cellophane-tape perianal swab method, as previously described [6], which was performed by parents between 07:30 and 09:30 in the morning, according to the instructions provided by the study researchers, and all samples were collected by the teachers at every kindergarten. Collected samples were then transported to the Department of Human Parasitology, Xinxiang Medical University, and examined under light microscope. The same detection procedure was performed on three consecutive days. If eggs were found, children were considered positive for *E. vermicularis* infection.

### Statistical analysis

Statistical analysis was performed using SPSS 20 software for Windows (SPSS Inc, Chicago, Illinois, USA). Statistical analyses of *E. vermicularis* prevalence for different variables were performed by χ^2^-test. The differences were considered statistically significant if *p* < 0.05.

## Results

In 2003, a total of 65 (12.75%) of the 510 samples were positive for *E. vermicularis* eggs. After 10 years, the egg positive rate significantly decreased to 5.13% (89/1734) in 2013 (Table 1).

**Table 1:** Egg positive rate of *Enterobius vermicularis* in children in studies in 2003 and 2013.

Year	No. examined	No. positive	Prevalence (%)	*p*-value
2003	510	65	12.75	<0.001
2013	1734	89	5.13	

Table 2 shows the personal hygiene practices and other factors that may potentially be associated with the egg positive rates of *E. vermicularis*. No significant differences in egg positive rates according to gender were observed in 2003 or 2013. The egg positive rate in rural areas was only slightly higher than that in urban areas in both 2003 and 2013. However, this difference was not significant (*p* > 0.05).

**Table 2:** Results of univariate analysis of risk factors and *Enterobius vermicularis* infection in the study participants.

Variable	2003				2013			
	No. examined	No. positive	Prevalence (%)	*p*-value	No. examined	No. positive	Prevalence (%)	*p*-value
Gender								
Male	282	39	13.83	0.414	918	53	5.77	0.200
Female	228	26	11.40		816	36	4.41	
Age (years)								
2–4	367	40	10.90	0.045	894	26	2.91	< 0.001
5–6	143	25	17.48		840	63	7.50	
Area								
Urban	253	30	11.86	0.551	994	49	4.93	0.657
Rural	257	35	13.62		740	40	5.41	
Hand washing before eating								
Yes	238	22	9.24	0.027	1156	41	3.55	< 0.001
No	272	43	15.81		578	48	8.30	
Sucking fingers or toys								
Yes	381	53	13.91	0.175	981	63	6.42	0.005
No	129	12	9.30		753	26	3.45	
Scratching around the anus								
Yes	287	41	14.29	0.237	611	52	8.51	< 0.001
No	223	24	10.76		1123	37	3.29	

In 2003, the egg positive rate among 5 to 6-year-old children (17.48%, 25/143) was significantly higher than that among 2 to 4-year-old children (10.90%, 40/367, *p* < 0.05). Similarly, the egg positive rate among 5 to 6-year-old children was also significantly higher than that among 2 to 4-year-old children (7.50% vs. 2.91%, *p* < 0.001) in 2013 (Table 2).

Among selected personal hygiene factors, hand washing before eating, sucking fingers or toys, and scratching around the anus, were all associated with enterobiasis. Children who wash their hands before eating had lower egg positive rates for *E. vermicularis* than those who did not (*p* < 0.05). Children with the habit of sucking fingers or toys were more commonly infected by *E. vermicularis* than those who did not have this habit; this result was significant in 2013 (*p* < 0.01) but not in 2003 (*p* > 0.05). Similar results were found in the children with the habit of scratching around the anus or not.

## Discussion

Although enterobiasis is generally considered to be a nuisance rather than a serious disease, the level of morbidity is significant, particularly in children.

This study found that the overall prevalence of *E. vermicularis* infection in preschool children was 12.75% in 2003 and 5.13% in 2013 in Xinxiang. With the implementation of the National major parasitic diseases prevention and control project (2006–2015) and the National soil source nematode disease prevention and control project (2006–2015) (National Health and Family Planning Commission [NHFPC] of the People’s Republic of China), awareness levels of enterobiasis among children’s carers, such as teachers and parents, as well as health behavior training rates of children, have clearly increased. In addition, almost full coverage of non-hazardous sanitary toilets in the countryside has been achieved. Moreover, the average number of students in each class of the kindergarten decreased from 35 to 30. The infrastructures of kindergartens such as toilets and playground equipment are much cleaner and more spacious than 10 years ago. These changes might explain why the prevalence of *E. vermicularis* infection has decreased to a low level in Xinxiang.

In the present study, the egg positive rate for *E. vermicularis* among 5 to 6-year-old children was also significantly higher than that among 2 to 4-year-old children in both 2003 and 2013. Five to 6-year-old children might have more frequent contact in kindergartens than children 2–4 years of age. The egg positive rate for *E. vermicularis* was also found to significantly increase with age, as previously reported [6, 7]. With regard to gender, no significant statistical difference was observed between boys and girls, which was consistent with other reports [12, 15, 18].

According to previous reports regarding risk factors for *E. vermicularis* infection, inadequate personal hygiene increased the risk of enterobiasis among primary school children [9, 11, 17]. *E. vermicularis* can be transmitted from person to person by contaminated hands, especially in crowded conditions [3]. Children who did not mention that they should wash their hands with soap before eating had higher concentrations of *E. vermicularis* eggs on their hands (*p* < 0.05) than those who did [3]. In the present study, children who wash their hands before eating had lower egg positive rates for *E. vermicularis* than those who did not. These studies highlight the need to teach children to get into the recommended habit of washing hands at school and at home, under the supervision of teachers and parents, respectively, to prevent transmission of *E. vermicularis.*

Furthermore, in our study, children with the habit of sucking fingers or toys showed higher rates of *E. vermicularis* infection than those who did not have this habit. This was consistent with previous reports [5, 9, 10]. For younger children, guardians should pay close attention to cleaning toys and prohibit children from sucking fingers or toys.

It is therefore necessary not only to carry out mass screening and regular whole group treatments for children, with correct medication methods, but also to develop pinworm eradication programs, such as health education programs on enterobiasis for parents, in order to control *E. vermicularis* infection in China.

In conclusion, the prevalence of *E. vermicularis* infection among preschool children decreased significantly over a period of 10 years in Xinxiang city, but *E. vermicularis* infection was still prevalent. Therefore, specific control measures are required to interrupt the transmission cycle of *E. vermiculari*s. Larger scale studies are required to establish the extent of the infection in other parts of China.

## Conflict of interest

The authors declare no conflict of interest in relation with this paper.
